# Functional and Aesthetic Restoration After Surgical Treatment of Oral Squamous Cell Carcinoma Using Radial Forearm Free Flap: Case Report

**DOI:** 10.3390/dj13110499

**Published:** 2025-10-28

**Authors:** Silviu Vultur, Dániel Száva, Alexandra Mihaela Stoica, Mara Vultur

**Affiliations:** 1 Department of Plastic, Aesthetic and Reconstructive Microsurgery, County Emergency Clinical Hospital of Târgu Mureș, 540136 Târgu Mureș, Romania; silviu.vultur@yahoo.com (S.V.); nemesmara1997@gmail.com (M.V.); 2 Department of Oral and Maxillo Facial Surgery, George Emil Palade University of Medicine, Pharmacy, Science and Technology of Târgu Mureș, 540142 Târgu Mureș, Romania; daniel.szava@umfst.ro; 3 Department of Odontology and Oral Pathology, George Emil Palade University of Medicine, Pharmacy, Science and Technology of Târgu Mureș, 540139 Târgu Mureș, Romania

**Keywords:** oral squamous cell carcinoma, radial forearm free flap, microvascular reconstruction

## Abstract

**Background:** Oral squamous cell carcinoma (OSCC) is the most common malignancy of the oral cavity, often necessitating extensive surgical resection. Such interventions may result in complex intraoral defects requiring immediate reconstruction to restore function and aesthetics. **Objective:** This case report highlights the surgical management of a patient with OSCC involving the tongue, floor of the mouth and mandibular ridge, reconstructed using a radial forearm free flap (RFFF). Case report: A 51-year-old male with a history of heavy smoking presented with a necrotic lesion affecting the left mandibular alveolar ridge, floor of the mouth, and tongue. **Methods:** Histopathological examination confirmed a diagnosis of moderately differentiated keratinizing OSCC (G2). After oncologic resection and selective neck dissection, the defect was reconstructed using an RFFF harvested from the left forearm. The facial artery and anterior jugular vein served as recipient vessels for microvascular anastomosis. A split-thickness skin graft (STSG) was used to close the donor site. **Results:** The postoperative course was generally favorable. Minor complications, including a localized hematoma and neck wound dehiscence, were conservatively managed. Functional outcomes such as oral intake and wrist mobility were successfully restored with rehabilitation. The RFFF provided durable, well-vascularized coverage over exposed mandibular bone, critical for minimizing the risk of osteoradionecrosis in the context of planned adjuvant radiotherapy. **Conclusions:** The radial forearm free flap remains a reliable reconstructive option for complex oral defects post-OSCC resection. Multidisciplinary collaboration and meticulous surgical technique are essential to achieve optimal oncologic, functional, and aesthetic outcomes.

## 1. Introduction

Oral squamous cell carcinoma (OSCC) constitutes the most common malignancy of the oral cavity, representing approximately 90–95% of all oral cancers worldwide, with incidence rates continuing to rise, particularly in populations with high exposure to established risk factors such as tobacco use, alcohol consumption, betel quid chewing, and poor oral hygiene. Despite improvements in diagnostic capabilities and therapeutic modalities, OSCC is frequently diagnosed at advanced clinical stages due to the asymptomatic nature of early lesions and delayed patient presentation [1,2].

The primary treatment modality for OSCC remains surgical resection with histologically negative margins. However, the anatomical complexity of the oral cavity, particularly in subsites such as the floor of the mouth, lateral tongue, and mandibular alveolar ridge, often necessitates extensive composite resections involving soft tissue, mucosa, and occasionally bone. These resections result in complex three-dimensional defects that compromise vital functions including deglutition, articulation, mastication, and airway protection, and may also lead to significant facial disfigurement. In such cases, functional and aesthetic rehabilitation is integral to optimizing postoperative recovery and improving the patient’s overall quality of life [3].

Microvascular free tissue transfer has revolutionized head and neck reconstruction, offering a reliable solution for the restoration of form and function following ablative surgery. Among the various reconstructive options available, the radial forearm free flap (RFFF) is widely regarded as a workhorse flap for intraoral and oropharyngeal reconstruction. It provides thin, pliable, and well-vascularized tissue with a long, consistent vascular pedicle, allowing for tension-free microvascular anastomoses even in deep or restricted recipient sites [4]. The flap’s versatility and favorable donor site morbidity profile have made it a preferred choice in cases requiring immediate reconstruction following OSCC excision. Furthermore, the use of vascularized free tissue has been shown to enhance wound healing, decrease complication rates, and support long-term functional recovery, particularly in high-risk surgical beds [5,6].

This case report presents the comprehensive surgical management of a patient with a moderately differentiated OSCC involving the floor of the mouth, left mandibular alveolar ridge, and adjacent tongue, emphasizing the critical role of multidisciplinary planning, meticulous microsurgical technique, and the use of a radial forearm free flap for immediate reconstruction. The report details the preoperative assessment, operative strategy, reconstructive approach, and postoperative outcomes, contributing to the growing body of evidence supporting the efficacy and safety of the RFFF in complex head and neck oncologic surgery [7,8,9].

## 2. Case Presentation

This study was conducted in accordance with the ethical principles set forth in the Declaration of Helsinki (1997 revision). Prior to initiation of the surgical intervention, the study protocol received approval from the Medical Ethics Committee for the Clinical Study of Medicine within the SCJU Târgu Mureș no. Ad.19418 of 23.07. Written informed consent was obtained from the patient after providing a comprehensive explanation of the procedure, potential risks, and expected outcomes (A/no.215).

A 51-year-old male patient from Târgu Mureș, Romania, initially presented to his general dentist seeking comprehensive dental care. The patient, who was fully edentulous, reported progressive ill-fitting of his dentures and painful mastication, with symptom onset approximately six months prior to presentation. Intraoral clinical examination revealed an ulcerated lesion with a necrotic base, irregular margins, and overlying fibrinous exudate, located along the left mandibular alveolar ridge and floor of the mouth, extending to the ventrolateral surface of the tongue.

**Figure 1:**
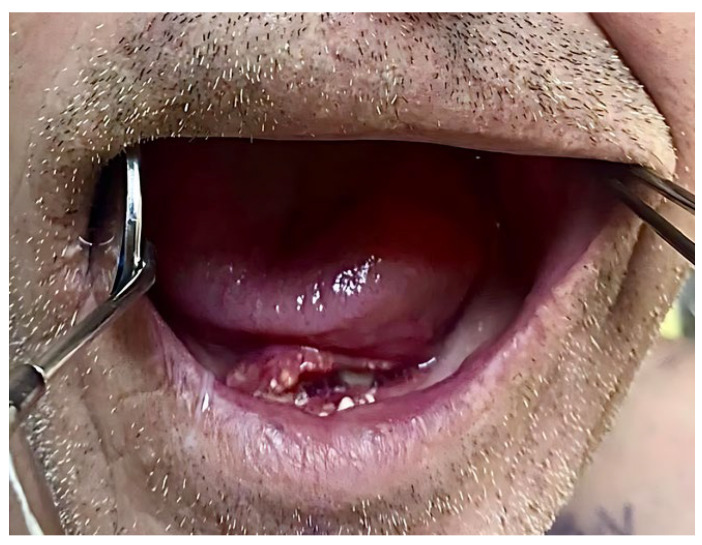
Clinical aspect of the intraoral lesion.

The surrounding mucosa was erythematous and indurated, with firm underlying tissues suggestive of deep infiltration, involving multiple anatomical subsites without overt bleeding or purulence, findings which raised strong clinical suspicion for an underlying malignant pathology (Figure 1).

At the time of the initial clinical evaluation, the patient reported a past medical history significant for chronic bronchitis, mild mitral valve regurgitation, and sinus tachycardia. He also disclosed a longstanding history of heavy tobacco use, with an estimated consumption exceeding 20 cigarettes per day over the past 30 years. The patient presented with a body weight of 67 kg, was alert, cooperative, and fully oriented to time, place, and person, indicating preserved cognitive function. No prior oncological diagnoses or relevant surgical interventions were documented.

**Figure 2:**
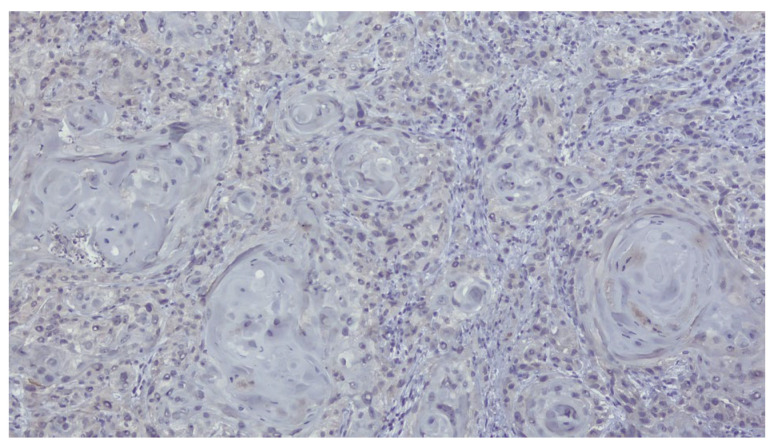
Biopsy-immunohistochemical reaction-p16 marker-negative.

The patient was referred to the Department of Oral and Maxillofacial Surgery for further evaluation and management. Following an outpatient consultation and comprehensive clinical examination, no palpable cervical lymphadenopathy was identified on physical assessment. Incisional biopsy of the oral lesion was performed under local anesthesia and the histopathological analysis of the biopsy specimen subsequently confirmed the diagnosis of moderately differentiated keratinizing squamous cell carcinoma grade II Immunohistochemical staining for p16 was also performed and showed a negative result, indicating that the lesion was not HPV-related but more consistent with a conventional, tobacco-associated carcinogenesis pathway (Figure 2).

As part of the comprehensive preoperative assessment, the patient underwent routine laboratory testing, including hematological and biochemical panels, alongside specialized cardiological and pulmonary evaluations. Diagnostic imaging included chest radiography, contrast-enhanced computed tomography (CECT) of the head and neck region, as well as the chest and bilateral upper limb computed tomography angiography (CTA) to evaluate systemic fitness for surgery and to identify any anatomical or vascular contraindications to radial forearm free flap harvest.

Preoperative laboratory investigations revealed isolated biochemical abnormalities, with no significant hematological deviations. Specifically, serum sodium and urea levels were mildly decreased seven days prior to surgery (134 mmol/L and 14 mg/dL, respectively) and remained below reference thresholds on the day before the procedure (131 mmol/L and 10 mg/dL, respectively). In addition, a slight elevation in serum potassium was noted preoperatively, with a recorded value of 5.24 mmol/L.

Cardiological assessment revealed mild mitral valve regurgitation and sinus tachycardia, with preserved left ventricular function and no evidence of ischemic changes or arrhythmias that would contraindicate major surgical intervention.

The chest radiograph demonstrated accentuated interstitial markings in the left infrahilar region, raising suspicion for underlying bronchiectasis, along with multiple diffuse fibrotic bands.

Additionally, two peripheral basal opacities were identified in the right lung, projected over the seventh costal arches, findings suggestive of residual changes secondary to a prior post-traumatic rib fracture with associated callus formation.

**Figure 3:**
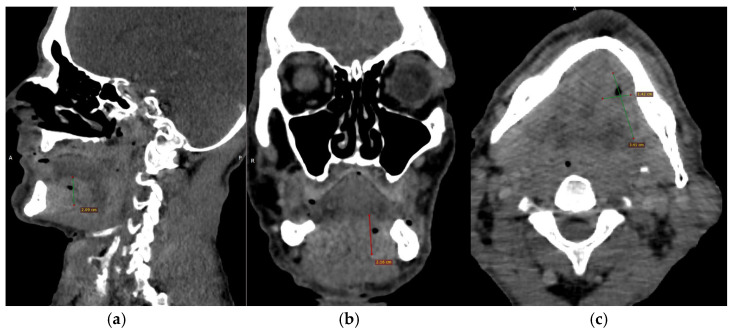
(**a**–**c**) CECT images (**a**) Axial section showing a heterodense lesion in the left sublingual space with central necrosis and peripheral enhancement. (**b**) Coronal section demonstrating the lesion confined to the left side without crossing the midline. (**c**) Sagittal section illustrating the depth of infiltration (18 mm) without evidence of mandibular bone involvement.

**Figure 4:**
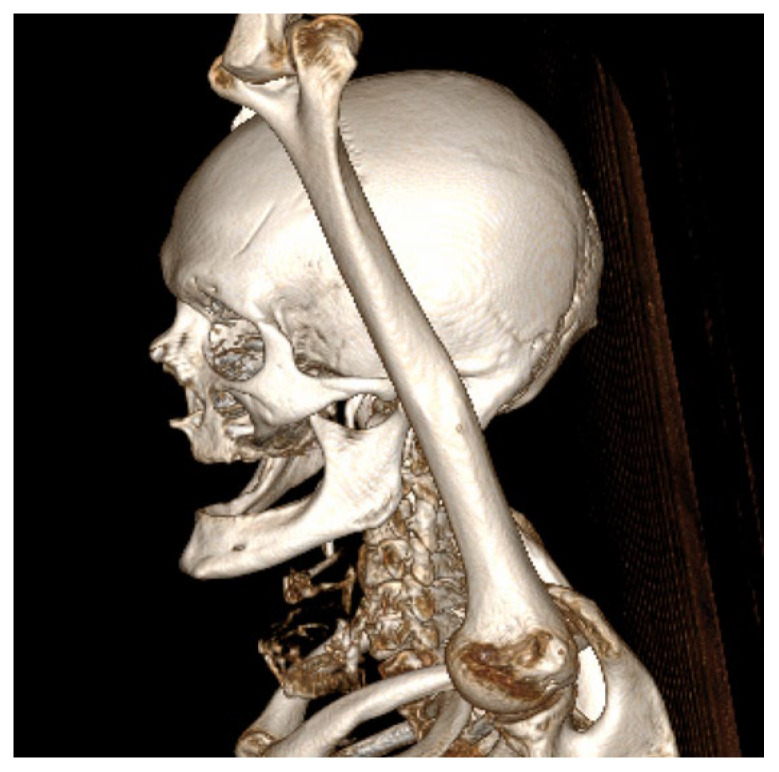
Three-dimensional CT reconstruction in left lateral view demonstrating the craniofacial skeleton, cervical vertebrae, and upper thoracic cage with no bone destruction.

CECT of the oral and submandibular region revealed a heterodense lesion within the left sublingual space, measuring approximately 34 × 13 × 20 mm, with a depth of 18 mm (Figure 3). The lesion exhibited fluid content with small central air inclusions and peripheral contrast enhancement, without evidence of adjacent osseous destruction (Figure 4).

It remained confined to the left side, without crossing the midline. The radiologic appearance was suggestive of a necrotic neoplasm or, less likely, an infectious etiology. Additional findings included a few punctate calcifications within the left parotid gland and an associated 4 mm hypodense area. A well-circumscribed, cystic-appearing lesion measuring 15 mm was also noted in the left subclavicular region, radiologically consistent with a possible lymphangioma. The carotid-jugular vascular axis was patent bilaterally with preserved caliber, although parietal calcifications were observed at the carotid bifurcations on both sides.

**Figure 5:**
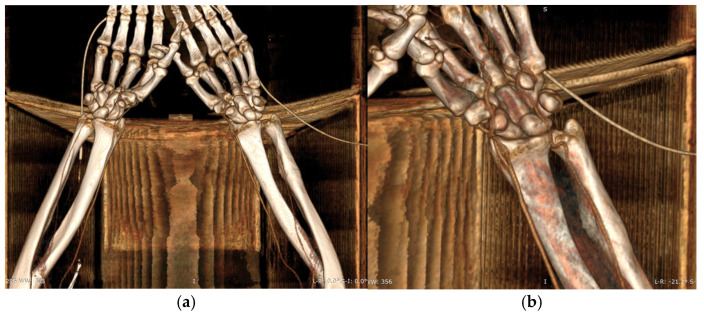
(**a**,**b**). CTA of upper limbs (**a**). CTA of the left upper limb (**b**).

CTA of the left upper limb demonstrated a fully patent arterial axis with preserved caliber and no pathological or anatomical abnormalities throughout the vascular course. The venous system was likewise patent, symmetrically configured, and of normal caliber bilaterally. No acute or suspicious alterations were noted in the soft tissues of the deltoid, axillary, brachial, ulnar, or forearm compartments on either side. Additionally, there were no radiologic signs of acute or chronic bone lesions within the examined regions. CTA of the right upper limb revealed a similarly patent and anatomically preserved arterial axis. However, a small, calcified atheromatous plaque was observed approximately 1 cm distal to the brachial artery bifurcation, producing only a minimal, clinically insignificant degree of luminal narrowing (Figure 5a,b).

Following completion of the preoperative workup, the patient provided written informed consent for surgical intervention. Given the extent of the tumor and the imperative for reliable functional reconstruction post-resection, a radial forearm fasciocutaneous free flap was selected as the preferred method of reconstruction via free tissue transfer.

An additional critical factor influencing the choice of reconstructive technique was the high likelihood that the patient would require postoperative adjuvant radiotherapy. In such cases, achieving robust, well-vascularized soft tissue coverage, particularly over exposed mandibular bone, is essential to reduce the risk of osteoradionecrosis, a severe and often debilitating complication.

Inadequate mucosal or soft tissue coverage in irradiated fields has been strongly associated with delayed wound healing, pathological bone exposure, chronic infection, and progressive necrosis of the mandible. The radial forearm free flap in this case offers reliable perfusion, pliability, and thin tissue characteristics, making it particularly suited for resurfacing intraoral defects and protecting osseous structures from radiation-induced ischemic damage.

Given the complexity of the case and the anticipated duration of the surgical intervention, a multidisciplinary team approach was adopted to optimize operative efficiency and patient safety.

Two oral and maxillofacial surgeons performed the tumor resection and left-sided cervical lymphadenectomy, while a team of two plastic and reconstructive surgeons was responsible for the harvesting and preparation of the radial forearm free flap.

This collaborative strategy allowed simultaneous execution of ablative and reconstructive phases, thereby minimizing total operative time under general anesthesia and ensuring each surgical component was executed within the respective domain of subspecialty expertise.

The surgical procedure required the use of a dedicated microsurgical instrument set, including Keeler Supervu Vu SL HI resolution Galilean System 3.0x (Windsor, Berkshire, United Kingdom) magnification surgical loupes, medium 20 cm hemoclip applicators (DeBakey Cross-Action Bulldog Clamp (Teleflex Incorporated, Pennsylvania, United States), standard dissection tools such as Mayo scissors, and bipolar cautery forceps. To ensure a bloodless operative field during flap harvesting, an 18-inch pneumatic tourniquet cuff was applied in conjunction with Esmarch bandage exsanguination. A dermatome was also prepared for the procurement of a split-thickness skin graft, which was later used for closure of the forearm donor site. Serodrain catetere Redon CH8 (Écouen, France) closed-suction drainage systems were placed in both the donor and recipient sites to facilitate postoperative fluid evacuation and promote optimal wound healing.

The initial operative stage, designated as the septic phase, was performed by the oral and maxillofacial surgery team and involved tumor excision within the oral cavity. The patient was positioned supine on the operating table and underwent general anesthesia with nasotracheal intubation to facilitate intraoral access. Given the anticipated duration of the procedure, a urethrovesical catheter was placed following induction to ensure intraoperative urinary monitoring and bladder decompression.

**Figure 6:**
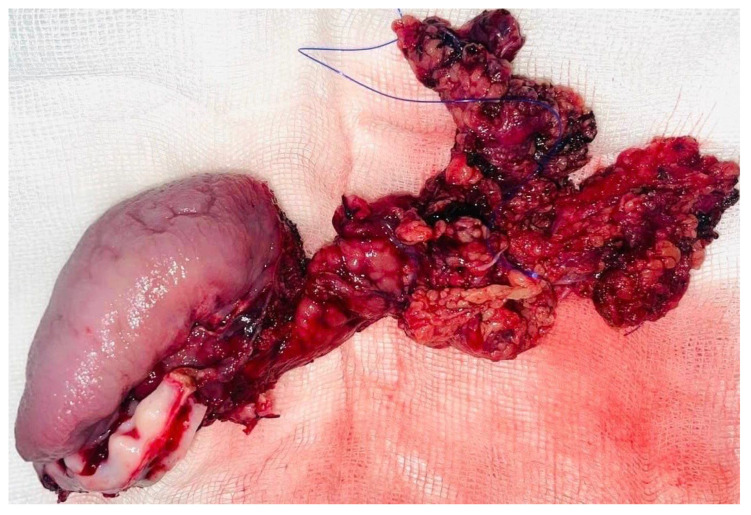
En bloc excision of the tumor, left submandibular salivary gland and lymph nodes.

A selective neck dissection was undertaken, comprising en bloc excision of the left submandibular, submental, upper jugular, upper spinal accessory, and middle jugular lymph node levels (Figure 6). Concurrently, the left submandibular gland was excised, with careful preservation of the spinal accessory nerve to minimize postoperative functional morbidity.

Although the spinal accessory nerve was meticulously preserved during the selective neck dissection, postoperative functional morbidity may still occur due to factors such as intraoperative traction, ischemic insult to the vasa nervorum, perineural fibrosis, or neuropraxia, all of which can compromise neuromuscular integrity and lead to clinical manifestations including shoulder dysfunction, limited range of motion, trapezius muscle weakness, or chronic pain.

**Figure 7:**
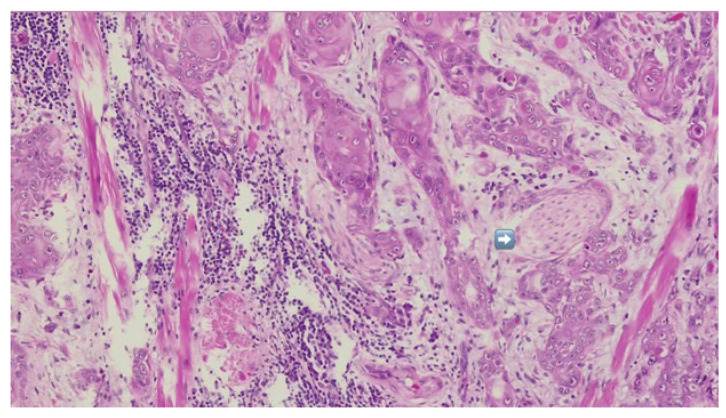
HE-infiltrative squamous cell carcinoma with peri-neural invasion—indicated by blue arrow.

Tumor resection was subsequently performed with a macroscopic oncologic safety margin of approximately 1 cm. The primary surgical specimen, including the neoplasm, associated lymph node groups, and the submandibular salivary gland, was removed en bloc via a transoral approach. The specimen was immediately fixed in 10% buffered formalin and submitted for definitive histopathological evaluation (Figure 7).

Definitive histopathological analysis of the surgical specimen confirmed a moderately differentiated keratinizing squamous cell carcinoma with evidence of perineural invasion (Figure 7). All surgical margins were free of tumor infiltration (R0 resection). Examination of the lymph nodes removed during selective neck dissection revealed no metastatic involvement (0 nodes). No evidence of distant metastasis was identified on preoperative imaging or postoperative staging, confirming a final pathological stage of pT3N0M0 (Stage III) according to the AJCC 8th edition.

To ensure oncologic adequacy, intraoperative frozen section analysis was conducted at the resection margins and confirmed complete excision with negative tumor involvement. Upon completion of the septic phase, the surgical field was re-prepared and re-draped in accordance with aseptic protocols to proceed with the reconstructive phase.

The mucosal and soft tissue defect resulting from tumor resection, which included a left longitudinal hemiglossectomy, left floor and excision of the alveolar mucosa at the level of edentulous ridge of the left mandibular body, necessitated the adaptation of a surgical technique capable of covering the entire defect.

**Figure 8:**
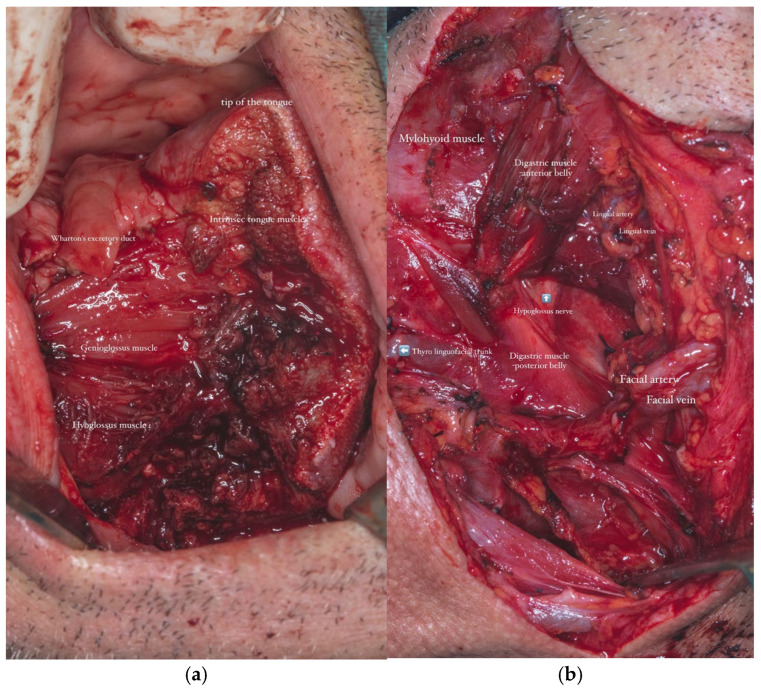
(**a**,**b**). Intraoperative imagine-tongue (**a**). Intraoperative imagine-neck dissection-dissection–the tiro-linguo-facial trunk and hypoglossus nerve showed by blue arrows (**b**).

In parallel, dissection and identification of the left facial artery and the anterior jugular vein were performed, as these vessels were selected as the recipient sites for the subsequent microvascular anastomoses during the reconstructive phase. The chosen tissue needed to closely approximate the characteristics of the native tissues in order to ensure optimal functional and aesthetic outcomes (Figure 8a,b).

During the preoperative evaluation, the left forearm, designated as the donor site, was thoroughly assessed and found to have no history of trauma, previous surgical intervention, or vascular compromise. The patient’s right-handed dominance, along with the presence of a calcified atheromatous plaque near the brachial artery bifurcation on the right side as identified on CTA, further supported the selection of the left forearm as the optimal donor site. At that time, collateral circulation was assessed with the Allen test, which confirmed adequate perfusion via the ulnar artery.

On the morning of surgery, the Allen test was repeated with consistent results, and the nursing team was instructed to avoid placement of intravenous lines or arterial catheters in the selected donor forearm in preparation for flap harvest.

Additionally, both the left forearm and the anterolateral aspect of the left thigh—intended as the donor site for the split-thickness skin graft were shaved preoperatively to optimize tissue harvest conditions. The radial forearm flap derives its arterial supply from the radial artery, while venous drainage is achieved via dual systems: the superficial venous system, which converges into the cephalic vein, and the deep venous system, which drains through the paired veins accompanying the radial artery.

**Figure 9:**
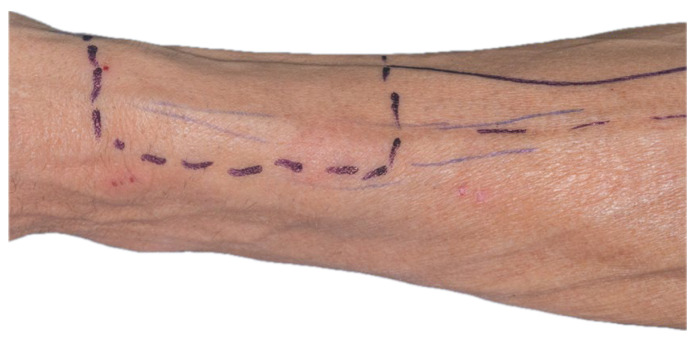
Forearm guidelines of anatomical landmarks.

The size and contour of the flap were delineated intraoperatively using a sterile surgical marker, guided by the dimensions of the composite defect involving the floor of the mouth and lingual region, which measured approximately 8 × 5.5 cm. A vascular pedicle length of 13 cm was determined as necessary to facilitate tension-free tunneling and microvascular anastomoses within the cervical region (Figure 9).

Anatomical landmarks were employed to precisely define the flap boundaries. The distal limit was aligned with the proximal flexion crease of the left radiocarpal joint. The ulnar border was marked medial to the projected trajectory of the palmaris longus tendon, while the radial boundary was set lateral to the anticipated path of the cephalic vein. These landmarks established the cutaneous paddle dimensions at approximately 5.5 cm in width and 8 cm in length. The radial artery served as the principal vascular axis for flap perfusion. To initiate pedicle dissection, an S-shaped incision was made along the anterior surface of the left forearm, allowing optimal exposure of the underlying vascular structures.

Prior to initiating the skin incision, exsanguination of the left hand and forearm was achieved using an Esmarch bandage, followed by inflation of a pneumatic tourniquet to a pressure approximately 100 mmHg above the patient’s systolic blood pressure to maintain a bloodless operative field. The upper limb was positioned in supination on the operating table, with the shoulder abducted to 90 degrees and the elbow fully extended to facilitate optimal surgical access.

**Figure 10:**
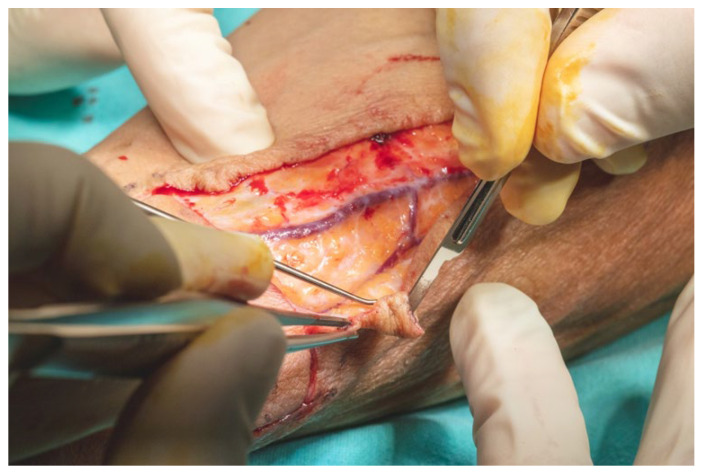
Forearm skin incision was made through the epidermis and dermis.

The skin incision was made through the epidermis and dermis in accordance with the preoperative markings, beginning at the ulnar margin of the designated flap area. The flap was elevated in a retrograde fashion under 3.5× magnification using surgical loupes. Dissection was carried out meticulously with fine dissection scissors, a No. 15 scalpel blade, and bipolar cautery forceps. Hemostasis was secured using 3.0 resorbable multifilament ligatures throughout the procedure to minimize bleeding and preserve tissue integrity (Figure 10).

**Figure 11:**
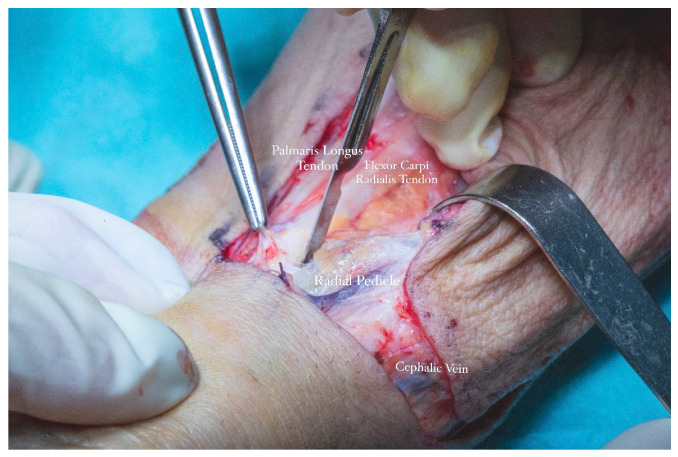
Forearm flap dissection revealing Palmaris Longus Tendon, Flexor Carpi Radialis Tendon, Radial Vascular Pedicle and Cephalic Vein.

Flap dissection was initiated in the suprafascial plane at the ulnar margin, progressing until identification of the flexor carpi radialis tendon, due to the presence of the superficial branch of the radial nerve and lateral antecubital nerve, which lies in the suprafascial plane (Figure 11). Once the nerve was identified, the dissection proceeded in the subfascial plane. Identification of the radial neurovascular bundle at the distal margin of the flap was a critical step. Following dissection of the skin paddle, the tourniquet was deflated, and the radial neurovascular pedicle was ligated and divided at the distal third of the left forearm.

**Figure 12:**
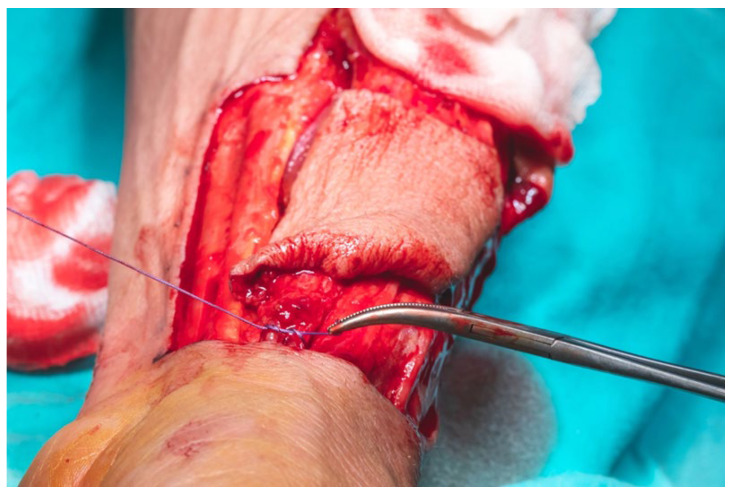
Ligation of the radial artery at the distal edge of the flap after the tourniquet was deflated.

Prior to ligation of the radial artery at the distal edge of the flap, hand perfusion was assessed by deflating the tourniquet and temporarily clamping the radial artery with a vascular clamp. This confirmed a positive Allen test. No signs of acute hand ischemia were observed, and capillary refill time was approximately 4 s (Figure 12).

**Figure 13:**
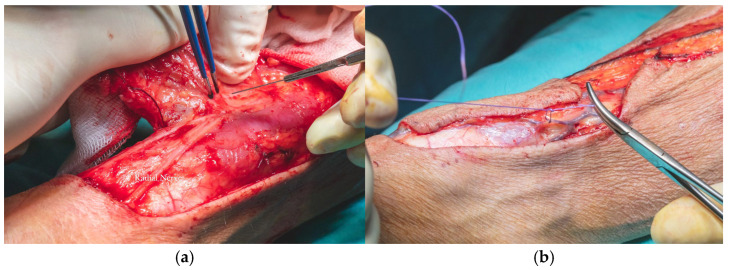
(**a**,**b**). Radial Nerve preservation and osseous perforators cauterization (**a**). Cephalic tributary veins ligaturing (**b**).

Meticulous care was taken during flap dissection to avoid injury to the paratenon overlying the flexor tendons, as preservation of this structure is essential for proper integration of the skin graft and for preventing postoperative cutaneous necrosis at the donor site. At the level of the wrist crease, the radial artery gave off multiple osseous perforators, which were identified and subsequently cauterized or ligated to maintain hemostasis. This anatomical feature also underscored the flap’s potential versatility, as it allows for harvest with an osseous component when bony reconstruction is required (Figure 13a,b).

**Figure 14:**
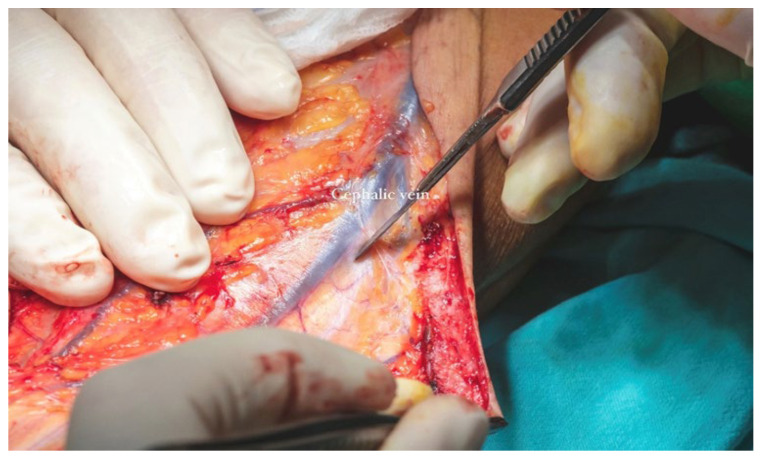
Cephalic vein incorporation within the flap design.

The cephalic vein was incorporated within the flap design and was therefore carefully dissected and ligated at the distal margin of the skin paddle. The S-shaped incision on the anterior aspect of the forearm extended proximally from the edge of the flap to the level of the elbow crease. In this region, dissection was performed in the suprafascial plane to preserve the integrity of the cephalic vein. The vein was meticulously mobilized, and its tributaries were ligated (Figure 13b) along a length of approximately 12–13 cm to ensure adequate pedicle length for microvascular anastomosis (Figure 14).

**Figure 15:**
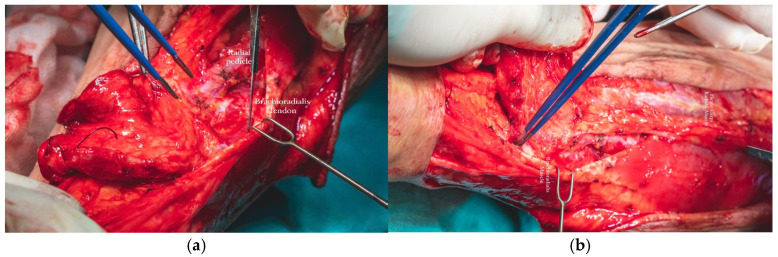
(**a**,**b**). Identification of the radial neurovascular bundle within the intermuscular septum (**a**,**b**).

The radial vascular bundle, beginning at the level of the radial pulse groove over the distal epiphysis of the radius, is positioned between the tendons of the brachioradialis and flexor carpi radialis muscles. To facilitate exposure, the brachioradialis muscle was gently retracted laterally. This maneuver allowed clear identification of the radial neurovascular bundle within the intermuscular septum separating the brachioradialis and flexor carpi radialis, enabling precise dissection and mobilization of the vascular pedicle (Figure 15a,b).

**Figure 16:**
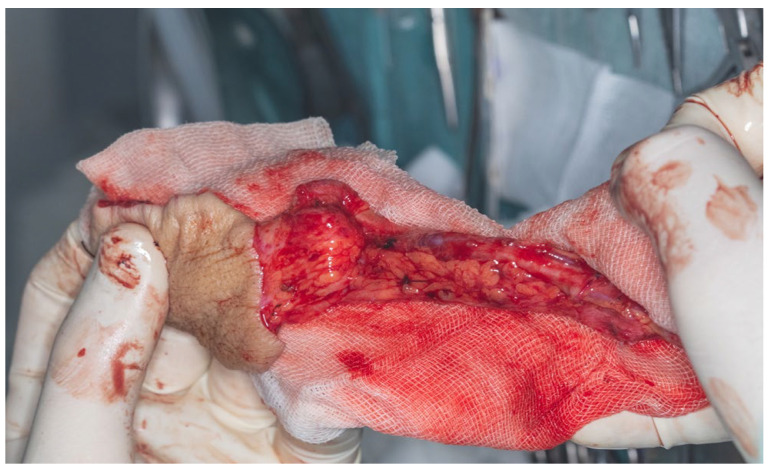
Final flap geometry with pedicle length of 13 cm and cephalic vein extended of 15 cm.

The total flap dissection time was 80 min, of which the initial 20 min were conducted in a bloodless field under pneumatic tourniquet control. Following completion of the pedicle dissection, the procedure transitioned to the microsurgical phase, which involved identification and preparation of the recipient cervical vessels. Given the notable discrepancy in vessel diameter between the cephalic vein (approximately 2.6 mm) and the left facial vein (approximately 5.2 mm), the anterior jugular vein on the left side was selected as an alternative recipient vein due to its more compatible caliber, measuring approximately 3 mm. After successful exposure and preparation of the recipient vessels, the radial vascular pedicle was ligated approximately 4 cm distal to the origin of the radial recurrent artery. The total pedicle length measured 13 cm, and the harvested cephalic vein extended approximately 15 cm (Figure 16).

To avoid the risk of pedicle injury, the flap was dissected and harvested with a small amount of surrounding adipose tissue and a portion of the intermuscular septum containing the radial pedicle.

**Figure 17:**
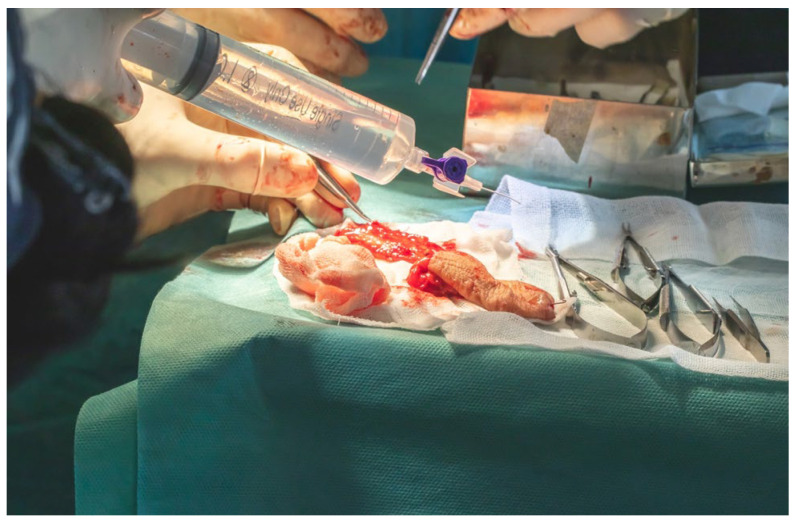
Arterial cannulation of the flap pedicle.

Arterial cannulation of the flap pedicle was performed using a 26-gauge cannula, through which a total of 50 mL of solution, comprising 45 mL of sterile normal saline and 5 mL (5000 IU) of fractionated heparin, was slowly irrigated. This intra-arterial flush ensured complete exsanguination of the flap, effectively minimizing the risk of intravascular coagulation during the ischemic interval prior to microvascular anastomosis (Figure 17).

The flap was secured at the lingual apex using a single 3.0 slowly resorbable multifilament ligature to prevent accidental displacement or avulsion into the lateral cervical region, thereby minimizing the risk of contaminating the operative field with oral cavity flora, despite the fact that the entire oral cavity had been chemically prepped preoperatively with a 7.5% Betadine solution. Primary end-to-end anastomosis was performed under 3.5× magnification loupes using interrupted 8.0 and 9.0 polypropylene monofilament sutures with round-bodied needles.

**Figure 18:**
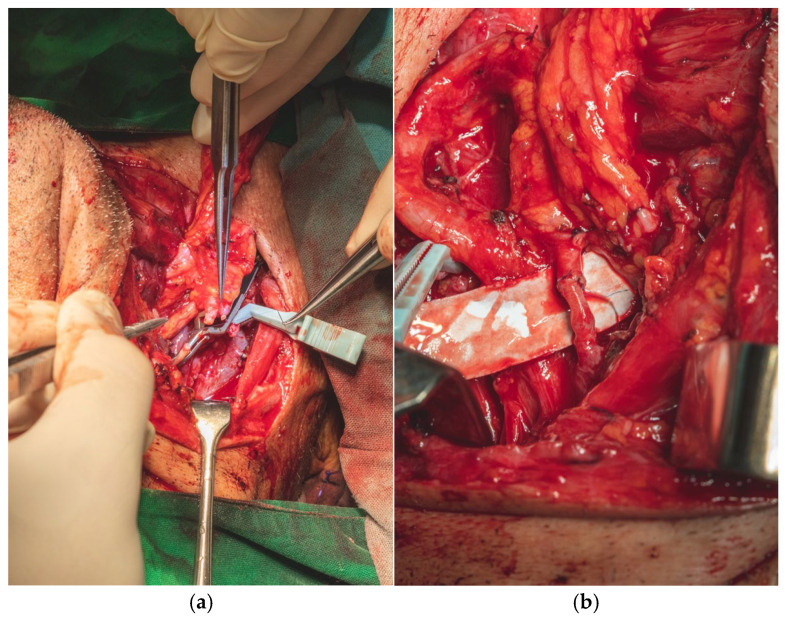
Intraoperative imagine of arterial anastomoses (**a**,**b**).

We performed all surgical procedures involving revascularization, reimplantation, or reconstruction with free flap transfer using a standardized microsurgical protocol. The arterial anastomosis was carried out first, followed by a waiting period of 5–10 min during which the flap is allowed to bleed. This step enables the assessment of blood flow restoration, confirmed by capillary refill and venous outflow from the flap (Figure 18a,b).

For the venous anastomosis, a primary end-to-end venorrhaphy, only interrupted 9.0 polypropylene monofilament sutures were used. Due to the direct inclusion of the cephalic vein within the cutaneous paddle of the flap, venous drainage was successfully achieved without any signs of venous congestion. Arterial inflow from the facial artery ensured adequate perfusion, with a capillary refill time of 3 s observed in the flap

**Figure 19:**
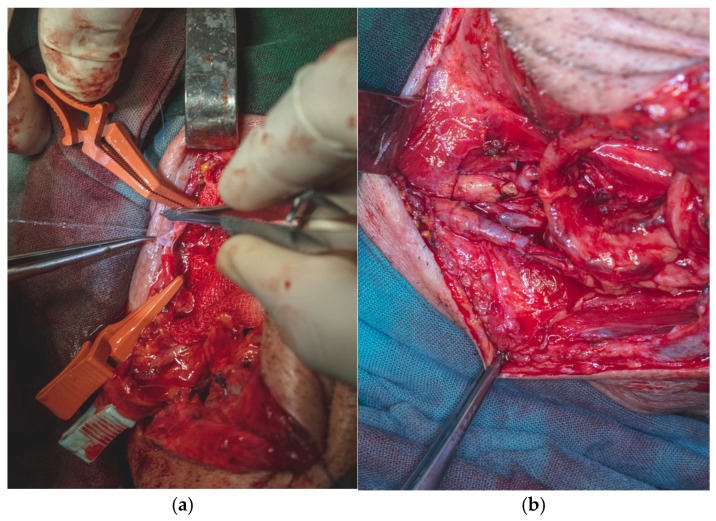
Intraoperative image of venous anastomoses (**a**,**b**).

Once the anastomoses were completed, we protected the arteriography and venography sites by securing the pedicle with 5.0 absorbable multifilament sutures to the mandibular periosteum on the inner surface of the mandibular body, at the point where the pedicle crosses the floor of the mouth to enter the oral cavity. At the level of the arterial and venous anastomoses, the pedicle was also anchored to the surrounding fascial structures (Figure 19a,b).

Fixation of the vascular pedicle to mobile anatomical structures such as the pharyngeal or laryngopharyngeal muscles was deliberately avoided, as postoperative extubation and the resumption of muscular activity could result in undue traction or potential disruption of the microvascular anastomoses. To mitigate this risk, the pedicle was intentionally harvested with an extended length of approximately 5 cm, allowing for greater flexibility, reduced tension, and enhanced safety during the postoperative recovery phase and early functional rehabilitation.

**Figure 20:**
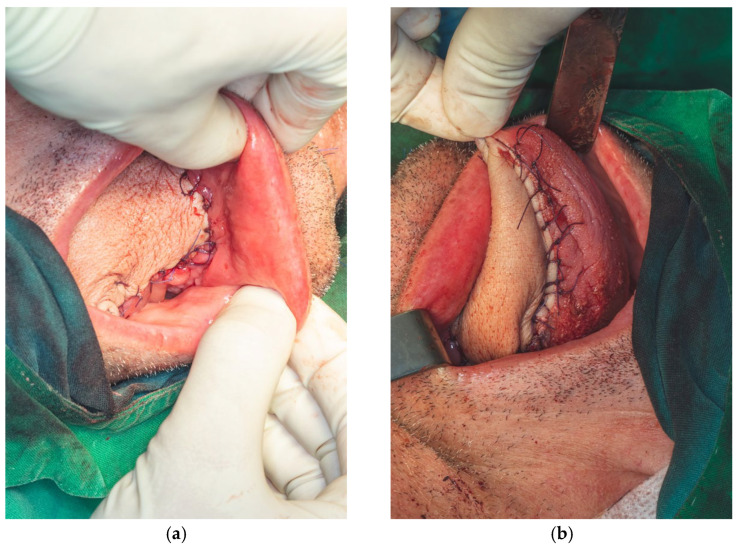
Wound closure of the oral floor (**a**). Tongue defect reconstruction (**b**).

Wound closure in the left submandibular and lateral cervical regions was performed in a layered fashion. The subcutaneous tissue was approximated using 4.0 absorbable multifilament sutures, while the skin was closed with interrupted 4.0 monofilament polypropylene sutures to ensure secure and precise apposition. Prior to final closure, a passive drainage tube was placed in a dependent position, distant from the site of vascular anastomoses, in order to minimize the risk of inadvertent pedicle traction or avulsion during postoperative drain removal (Figure 20a,b).

Closure of the donor site was carried out concurrently with the revascularization phase. Following meticulous hemostasis, a deep intermuscular suction drain was placed, with its distal tip positioned approximately 3 cm from the edge of the cutaneous defect to facilitate effective fluid evacuation. The antebrachial fascia was approximated using interrupted 3.0 and 4.0 absorbable multifilament sutures. A limited subcutaneous suture layer was employed where necessary, and skin closure was completed using an intradermal running suture with 4.0 monofilament polypropylene. To reduce tension along the suture line and support optimal healing, the wound was externally reinforced with adhesive strips.

**Figure 21:**
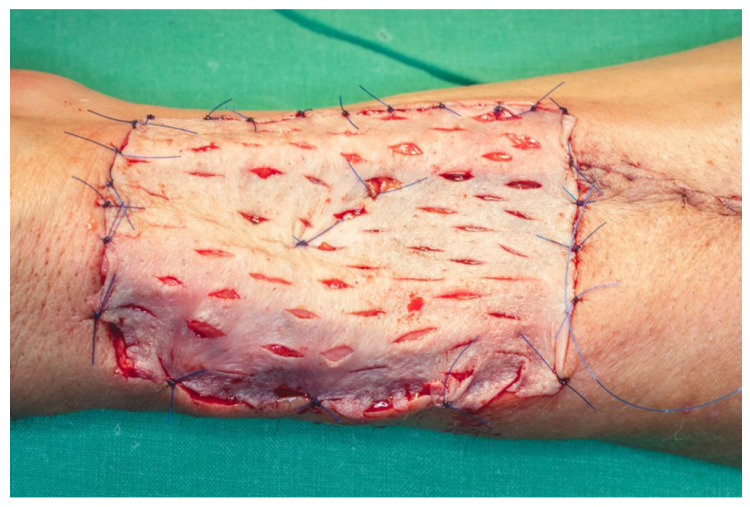
Cutaneous defect resurfaced using a split-thickness skin graft.

The cutaneous defect was resurfaced using a split-thickness skin graft (STSG), harvested with an electric dermatome from the anterolateral surface of the proximal third of the left thigh. The harvest site was selected to facilitate scar concealment. A medium-thickness graft was chosen, taking into account the relatively small surface area of the defect and the unavailability of a skin meshing device. Multiple drainage fenestrations were created in the graft using a No. 15 scalpel blade to permit egress of wound exudate and prevent hematoma formation beneath the graft, which could otherwise compromise graft viability. The graft was secured in place using 5.0 monofilament polypropylene sutures, and a tie-over dressing was applied to ensure even pressure distribution and optimal adherence of the graft to the wound bed (Figure 21).

Due to the anatomical location of the donor site, the left forearm was immobilized postoperatively in a dorsal rigid splint, with the limb positioned in a functional resting posture encompassing the forearm, palm, and digits. Final flap inset and contouring were performed following the application of sterile dressings to both the forearm and lateral cervical wound. The flap was secured in place using interrupted 3.0 non-absorbable multifilament sutures to ensure stability and optimal integration.

Given the presence of a mucosal defect with exposed mandibular bone, the adjacent buccal mucosa was carefully undermined and advanced to allow for tension-free approximation with the edge of the flap. Due to the minimal subcutaneous adipose tissue in this region, the flap thickness, measuring approximately 1 cm, allowed for effective coverage while preserving the ability to palpate the arterial pulse postoperatively.

After definitive surgical resection and histopathological evaluation of the specimen, the pathological staging (pTNM), which represents the stage established postoperatively from microscopic analysis, was confirmed as pT3N0M0 (Stage III).

Following extubation, a nasogastric feeding tube was placed to initiate enteral nutrition. The patient remained in stable general condition, with no immediate hemodynamic or respiratory complications.

Daily wound care with antiseptic solutions and routine dressing changes were performed. The tie-over dressing applied to the skin graft was removed on postoperative day 4. Drainage from the donor forearm site was monitored at 24 h intervals. The suction drain was removed on postoperative day 7 when the output had decreased to less than 30 mL per day. The passive cervical drain was removed on postoperative day 6. Both sutures and forearm immobilization were discontinued on postoperative day 10, after which external elastic compression was continued. Oral cavity sutures were removed on the same day.

Postoperative pharmacologic management included intravenous administration of amoxicillin-clavulanic acid (1.2 g, 3 times daily for 10 days), dexamethasone (8 mg/mL), initiated at 2 mL/day on the first postoperative day with gradual tapering until day 5, and subcutaneous anticoagulation with enoxaparin (40 mg Clexane^®^- Sanofi-Aventis, France twice daily for 10 days), beginning 8 h post-surgery. Additional supportive therapies included gastric protection, analgesics, and probiotics.

The patient developed a postoperative hematoma of approximately 100 mL on day 5, likely secondary to vascular trauma incurred during forceful removal of the passive cervical drain. A localized dehiscence of the lateral cervical wound was also noted, which subsequently healed by secondary intention within 18 days.

Other transient complications included left-sided cervical and supraclavicular edema, and regional ecchymosis. Temporary stiffness of the left radiocarpal joint and hand was observed following immobilization; however, full mobility of the wrist and digits was restored within one month through a structured physiotherapy program.

Oral candidiasis was diagnosed and successfully treated during hospitalization. The patient also reported a painful, electric shock-like tactile sensitivity at the flap donor site, attributed to incomplete coverage of the radial nerve by the overlying skin graft.

**Figure 22:**
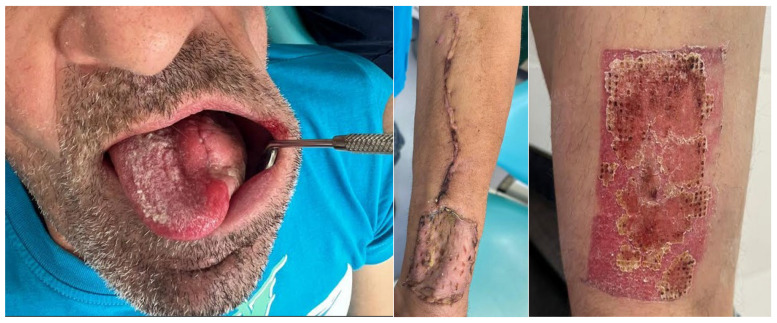
Post-operative clinical aspect at 14 days after sutures removal.

**Figure 23:**
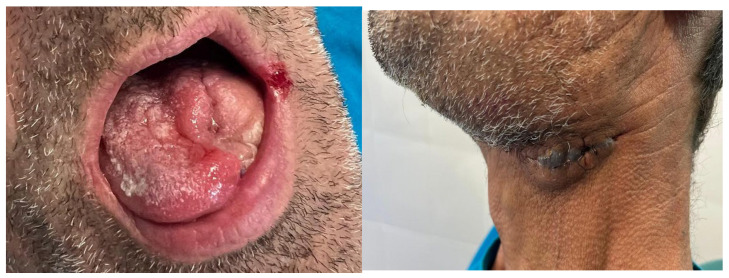
Post-operative clinical aspect at 14 days.

**Figure 24:**
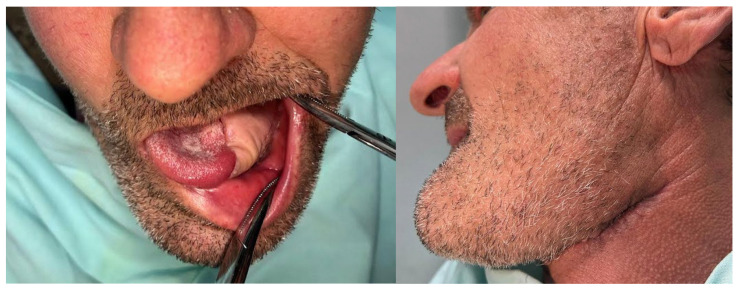
Post-operative clinical aspect at 2 months.

**Figure 25:**
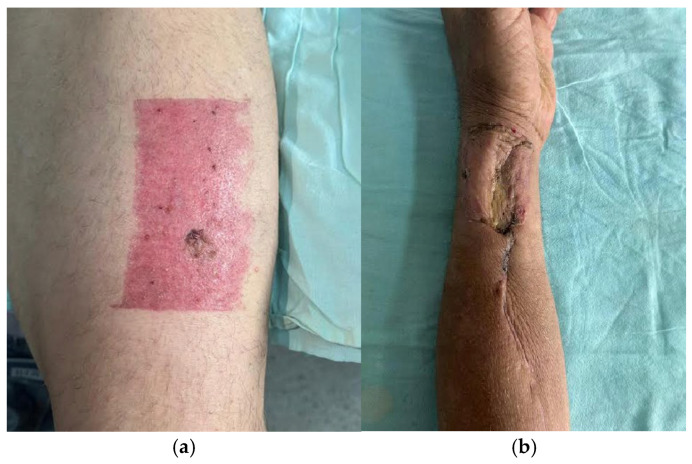
(**a,b**). (**a**). Clinical appearance of the donor bed from which the split-thickness skin graft was harvested, demonstrating appropriate healing at 2 months postoperatively. (**b**). Clinical aspect of the RAFF flap donor region, with complete (100%) integration of the overlying skin graft observed at 2 months postoperatively.

The following clinical photographs taken at 14 days and 3 months post-surgery are highlighting the progression of healing and final tissue integration (Figure 22, Figure 23, Figure 24 and Figure 25).

## 3. Discussion

The reconstructive demands following surgical management of OSCC are among the most complex in head and neck oncologic surgery. The primary objectives extend beyond oncological clearance and include restoration of function, preservation of oral competence, and re-establishment of aesthetic balance, goals that are particularly challenging when tumors involve the floor of the mouth, tongue, and mandibular ridge, as in the present case. The RFFF has been well-established as a first-line reconstructive modality in this context, owing to its distinctive anatomical and histological properties [10,11].

The thin, pliable, and highly vascularized nature of the RFFF offers an unparalleled advantage in intraoral reconstruction. Unlike bulkier myocutaneous or fasciocutaneous flaps, the RFFF conforms seamlessly to the dynamic contours of the oral cavity, enabling superior resurfacing of mobile structures such as the tongue and floor of mouth, while maintaining optimal mucosal pliability. Furthermore, its long and consistent vascular pedicle, along with dual venous drainage systems, facilitates microsurgical anastomoses even in anatomically restricted or compromised recipient beds [12].

From an oncologic standpoint, reliable flap perfusion plays a critical role in supporting the healing environment post-resection and in mitigating the deleterious effects of postoperative radiotherapy. This consideration was pivotal in flap selection for our patient, who was a strong candidate for adjuvant radiation [13].

Preoperative imaging and vascular evaluation, including contrast-enhanced CTA and the Allen test, ensured both flap viability and donor site safety. Notably, the presence of a calcified atheromatous plaque in the right upper limb steered flap harvest toward the left side, underscoring the importance of detailed vascular assessment. The Allen test remained a crucial bedside evaluation tool, supported by CTA findings, to confirm ulnar collateral circulation and minimize the risk of donor limb ischemia.

The execution of a dual-team surgical approach—combining oral and maxillofacial surgeons for tumor extirpation with reconstructive microsurgeons for flap harvest and anastomosis—provided procedural efficiency and enhanced patient safety. Simultaneous ablative and reconstructive phases reduced total operative time and optimized intraoperative resource utilization. Moreover, intraoperative frozen section margin assessment ensured oncologic adequacy while maintaining surgical flow.

Accurate staging is fundamental in the management of oral squamous cell carcinoma, as it guides both oncologic and reconstructive decision-making. In this context, documenting both the clinical (cTNM) and pathological (pTNM) staging provides a comprehensive overview of disease extent, acknowledging that discrepancies between preoperative imaging and intraoperative or histological findings may occur.

Incorporating both staging systems in case reporting not only strengthens the scientific rigor of documentation but also highlights the importance of contingency planning in reconstructive surgery, where intraoperative variations can influence flap design and overall treatment strategy.

Although donor site morbidity remains a consideration with RFFF, particularly related to sensory disturbance and graft integration, careful dissection techniques and protective strategies, such as paratenon preservation and the use of split-thickness skin grafts, mitigated complications. In our case, temporary dysesthesia at the donor site and mild wrist stiffness were managed conservatively, with full functional recovery achieved within one month [14].

Comparatively, alternative reconstructive options such as the anterolateral thigh flap or scapular/parascapular flaps (ALT) offer greater volume and versatility, particularly for composite defects involving bone or requiring bulk for external contouring. However, these flaps often involve longer operative times, more complex positioning, and greater donor site morbidity. In contrast, the RFFF maintains a superior profile for defects requiring thin, flexible coverage with a high degree of precision—particularly in elderly or comorbid patients [15].

The favourable clinical outcomes observed, evidenced by flap viability, early oral alimentation, and restored hand function are consistent with existing literature documenting flap success rates exceeding 95% and low complication profiles for RFFF in oral reconstruction. Furthermore, this case reinforces the role of functional reconstruction not merely as a surgical adjunct but as a determinant of postoperative quality of life, psychosocial well-being, and reintegration into daily activities [16].

Our approach aligns with current trends in reconstructive microsurgery, as reflected in a recent study by Accorona et al., which evaluated multiple cases of OSCC reconstruction. The authors found that RFFF offered superior intraoral adaptability, quicker postoperative oral function recovery, and fewer complications in irradiated fields compared to bulkier alternatives such as the ALT flap. Importantly, they noted that the RFFF was associated with higher patient-reported satisfaction regarding speech and deglutition, which supports the outcome observed in our patient [17].

Furthermore, a comparative meta-analysis by Patel et al. assessed a series of cases involving various flap techniques for oral cavity reconstruction. The study concluded that, while the ALT flap showed favorable donor site morbidity, the RFFF demonstrated better outcomes in terms of flap reliability, operative time, and suitability for small to medium-sized defects requiring mucosal flexibility. In particular, the RFFF was significantly associated with lower rates of postoperative fistula formation and better prosthetic dental rehabilitation in edentulous patients—both highly relevant to the clinical context of our case [18].

Well-vascularized free flaps are known to protect against radiation-induced complications, particularly osteoradionecrosis, by providing a stable and highly perfused soft tissue cover that promotes healing, reduces ischemia, and enhances tissue resistance in irradiated fields. The radial forearm free flap (RFFF) is especially advantageous in this context because its thin, pliable tissue conforms well to intraoral contours, ensuring tension-free coverage of exposed mandibular bone and mucosal defects. By re-establishing a robust vascular supply, the RFFF minimizes the risk of mucosal breakdown and subsequent bone exposure, both of which are precursors to radionecrosis. Compared with bulkier options such as the anterolateral thigh or pectoralis major flaps, the RFFF offers superior adaptability for small to medium intraoral defects, reduced donor site morbidity, and greater reliability in achieving watertight closure. Its consistent pedicle length and dual venous drainage also facilitate secure anastomosis, even in surgically altered or irradiated necks. Clinical evidence has demonstrated that patients reconstructed with RFFF experience fewer complications, including wound dehiscence, fistula formation, and delayed healing, when subjected to postoperative radiotherapy. These protective characteristics reinforce the RFFF as not only a reconstructive solution but also a preventive measure against the long-term sequelae of adjuvant radiation, thereby supporting improved functional recovery and quality of life [19]. Although our report documents the early and intermediate postoperative course up to 3 months, longer-term outcomes could not be assessed within the timeframe of this study. Based on existing evidence, patients reconstructed with a radial forearm free flap typically demonstrate durable flap viability, satisfactory long-term oral function (speech, deglutition, and mastication), and acceptable donor-site morbidity. The risk of flap failure beyond the first postoperative month is low, with reported success rates exceeding 95%. From an oncological perspective, the risk of local recurrence in oral squamous cell carcinoma remains highest within the first 2–3 years following resection, emphasizing the importance of rigorous surveillance. Functionally, early rehabilitation predicts favorable outcomes, and in our patient the return of oral intake and hand mobility within 3 months is encouraging. While adjuvant radiotherapy may pose risks of xerostomia, fibrosis, and osteoradionecrosis, the use of well-vascularized free tissue coverage, as achieved here, is protective against such complications [18,19].

Our case exemplifies the utility of the radial forearm free flap in addressing the multifaceted challenges of oral cavity reconstruction post-OSCC resection. The technique’s anatomical reliability, adaptability to complex defects, and radiotherapy resilience affirm its position as a gold standard in microsurgical head and neck reconstruction. Future studies may explore further refinements in donor site management, sensory recovery, and patient-reported outcome measures to optimize this already well-established approach.

## 4. Conclusions

The management of advanced oral squamous cell carcinoma presents a dual challenge: achieving complete oncologic resection while restoring function and aesthetics in a highly complex anatomical region. This case report demonstrates that the radial forearm free flap remains a versatile and reliable reconstructive option, particularly suited for intraoral defects involving the floor of the mouth, tongue, and mandibular ridge. A well-vascularized flap enables optimal resurfacing of intraoral structures, supports early functional recovery, and provides critical protection against radiation-induced complications, such as osteoradionecrosis.

Successful outcomes in such complex reconstructions depend not only on technical precision but also on comprehensive preoperative planning and interdisciplinary collaboration. The integration of oral and maxillofacial surgeons with plastic and reconstructive microsurgeons, combined with thorough vascular imaging and intraoperative microvascular expertise, proved essential in minimizing complications, optimizing surgical efficiency and obtaining satisfactory functional and aesthetic outcomes, reinforcing the flap’s value in head and neck oncologic surgery.

## Data Availability

The original contributions presented in this study are included in the article material. Further inquiries can be directed to the corresponding author.

## Abbreviations

The following abbreviations are used in this manuscript:

CECT	Contrast-Enhanced Computed Tomography
CTA	Computed Tomography Angiography
OSCC	Oral Squamous Cell Carcinoma
HE	Hematoxylin Eosin
STSG	Split-Thickness Skin Graft
ALT	Anterolateral Thigh Flap
cTNM	Clinical Tumor-Node-Metastasis staging; determined before surgery based on clinical examination and imaging findings
pTNM	Pathological Tumor-Node-Metastasis staging; determined after surgery based on histopathological examination of the resected specimen
